# Knowledge, attitude, and associated factors towards older people care among nurses working at public hospitals in West Shoa zone, Oromia region, Ethiopia

**DOI:** 10.1186/s12912-021-00774-1

**Published:** 2021-12-09

**Authors:** Firomsa Fita, Habtamu Sewunet Mekonnen, Helen Lamesgin Endalew, Abere Woretaw Azagew

**Affiliations:** 1 Ambo University referral hospital, Ambo, Ethiopia; 2 Department of Surgical Nursing, School of Nursing, College of Medicine and Health Sciences, University of Gondar, Gondar, Ethiopia

**Keywords:** Knowledge, Attitude, Nurses, Older people, West Shoa, Ethiopia

## Abstract

**Background:**

Nurses’ knowledge and attitude regarding the care of older people can have an impact on patient outcomes such as reduced length of hospital stays, reduced readmission rates, and increased patient and family satisfaction. However, evidence is scarce in Ethiopia, particularly in the study area. Therefore, the study aimed to assess the knowledge, attitude, and associated factors towards the care of older people among nurses working at public hospitals in West Shoa Zone, Ethiopia.

**Methods:**

Institutional based cross-sectional study was conducted from April1–30, 2021 among 423 nurses who were working in adult care units. Data were collected through a self-administered questionnaire. The sample was selected using simple random sampling. The logistic regression analysis model was fitted and the Adjusted Odds Ratio at 95% confidence interval was used. *P*-values less than or equal to 0.05 were considered statistically significant.

**Results:**

A total of 411 nurses participated in the study with a 97.16% response rate. The mean age of the participants was 29.11 (SD ± 3.84) years. The study showed that 37.2% (95% CI: 33, 42%) of the participants had good knowledge and 45.7% (95% CI: 40.9, 50.6%) had *a favorable attitude* toward the care of *older people. The significantly associated factors positively affected both the knowledge and the attitude of nurses.* Age greater than 30 years (AOR:2.37, 95% CI: 1.18, 4.75), experience greater than 5 years (3.00: 1.21, 7.41), being BSc degree holder and above (3.57: 1.40, 9.09), lived with older people (2.14: 1.34, 3.42), and nurses working in adult intensive care unit (3.03: 1.03, 8.91) were significantly associated with knowledge. Likewise, being female (2.04: 1.33, 3.12), being BSc degree holder and above (2.77: 1.35, 5.65), lived with older people (1.59: 1.03, 2.44), and care for older people (1.63: 1.06, 2.53) were significantly associated with attitude.

**Conclusion:**

In this study, less than half of the nurses had good knowledge and a favorable attitude towards the care of older people. Continuous professional development regarding the care of older people is important to enhance nurses’ knowledge and attitude.

## Background

Aging is a normal and irreversible phase in which the body undergoes physiological, chronological, psychological, and social changes [1]. Globally, the percentage of the population aged 60 years and above is increasing [2]. In 2017, there were about 962 million people aged 60 or above, accounting for 13% of the global population [3]. The number of older people in the world is estimated to be 1.4 billion in 2030 and 2.1 billion in 2050 and could grow to 3.1 billion in 2100. In Ethiopia, older people greater than 60 years represent 5% of the total population [4].

Nurses provide front-line health care for older people in a wide variety of settings, including preventive care in primary care offices and the community, acute care in hospitals, and long-term care in nursing homes and assisted living facilities [5]. The good knowledge and favorable attitude of nurses regarding the care of the older people can have a positive impact on patient outcomes, patient and family satisfaction, and can assist the caregivers in providing adequate care to older people [6, 7]. To provide high-quality older people care, nurses’ attitude toward the older people and their knowledge of the aging process are of paramount importance for practice and quality of care [8, 9]. Good knowledge and favorable attitude of nurses are important and regarded as a requirement for good quality health services for older people in a variety of different settings. Poor knowledge and unfavorable attitude towards the care of the older people can result in the prolonged hospitalization, unnecessary hospital readmission, and financial burdens and also increases the demands of hospital resources. It has a negative impact on the treatment outcomes as well [8, 10].

As studies in Iran, California, Bangladesh, Slovak Republic, India, and Zanzibar showed the magnitude of nurses’ knowledge about the older people care was 32.7, 80, 32.8, 91, 76.4, and 17.6% respectively [11–16]. In Iran, Portugal, Saudi Arabia, India, and Nepal, and Poland the magnitude of favorable attitude towards older people care was 9.8, 18.8, 65, 64.6, 50.3, and 36.9% respectively [11, 15, 17–19]. Regarding factors; age, gender, marital status, religion, year of work experience, level of education, working units, type of hospital, living with the older people, and experience in older people care were statistically significant variables associated with knowledge and attitude of nurses [11, 17, 19–24].

Despite the potential importance and crucial role of nurses in determining and delivering proper healthcare standards, and the quality of healthcare services, determination of the level of knowledge, attitude, and associated factors is necessary. However, evidences were scarce on nurses’ knowledge and attitude toward the care of the older people in Ethiopia, particularly in the study area. Therefore, the study aimed to assess knowledge, attitude, and associated factors towards care of older people among nurses working at public hospitals in West Shoa Zone, Ethiopia. The results of this study could be utilized as an input to nurses, hospital administration, regional health bureau, federal ministry of health, and other concerned bodies.

## Methods and materials

### Study setting

The study was conducted in West Shoa zone public hospitals. West Shoa zone is one of the zones of Oromia region in Ethiopia, *and it is located in the Western part of the country. Ambo town, which is located about 112 km from Addis Ababa, is the capital of West Shoa zone. The West Shoa zone has 8 public hospitals, 91 health centers, 526 health posts, 1 private higher clinic, 40 medium private clinics, 168 small clinics, 43 drug stores, 21 drug vendors, and 4 pharmacies. Within the zone, there are one teaching referral hospital, three general hospitals, and four primary hospitals. Ambo, Gedo, and Ginde beret are general hospitals, whereas Guder, Bako, Inchini, and Jeldu are primary hospitals. The study included all the public hospitals in the zone. The total number of nurses in West Shoa zone public hospitals was 701, of which 565 are working in adult care units/wards.*

### Study design and period

An institutional-based cross-sectional study was conducted *from April 01–30/2021.*

### Source and study populations

All nurses who were working at public hospitals in West Shoa zone, Ethiopia were the source population and **t**hose nurses who were working in adult care units at the public hospitals and found during the data collection period were the study population.

### Eligibility criteria

All nurses who were working in medical ward, surgical ward, operation room, emergency, ICU ward, gynecology ward, a psychiatric unit, optometric unit, dental clinic, medical OPD, surgical OPD, and nurses on medical and surgical chronic follow up clinics were included in the study. Nurses who were not available during data collection (on annual, maternal, sick leave, and training) were excluded.

### Sample size and sampling procedure

#### Sample size determination

The sample size was calculated using a single population proportion formula, considering the following assumptions: Confidence level (CI) =95%, Degree of precision (d) =0.05. The proportion (*p*) =50% (no similar study).

Using *n* = Z α/2) ^2^ p (1-p).

d^2^

*n* = (1.96)^2^ (0.5(1–0.5)/ (0.05)^2^ *n* = 384. By considering 10% non-response rate the final sample size was 423.

#### Sampling procedure and techniques

**Fig. 1:**
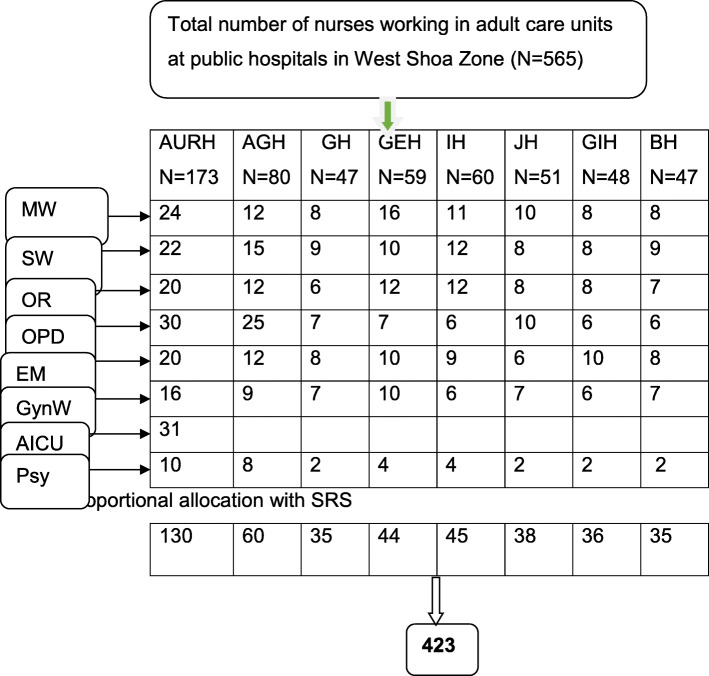
Schematic presentation of sampling procedure

All public hospitals in the West Shoa zone were included in the study. First, to get the sample size, all hospitals were included in the study. Secondly, the total calculated sample size of 423 nurses was proportionally allocated to each hospital based on the number of nurses in the adult units/wards. Finally, the simple random sampling method was used to select those proportionally allocated study participants. Figure 1.

#### Operational definition

##### Good knowledge

Respondents with a KOP-Q score of ≥75% were classified as having a good knowledge and those with score of < 75% were classified as having a poor knowledge [25].

##### Favorable attitude

Respondents with OPACS mean score of ≥3 classified as having a favorable attitude and those with mean score of <3were classified as having unfavorable attitude [25].

##### Older people

According to the UN definition, older people are those people whose age is 60 years and over [26]. The definition has gained acceptance in the Ethiopian context as it coincides with the country’s official retirement age [27].

##### Type of hospital

A type of facility that provides health care service at either primary, general, or referral level.

##### Working hours

The normal hours of work are 8 h a day and 39 h a week, according to the Ethiopian law Labor proclamation.

### Data collection tools and techniques

Data were collected through a self-administered questionnaire using the Knowledge of Older Patients Quiz and Older People in Acute Care Survey. The KOP-Q was developed and validated among 331 participants in the Netherlands and USA [28, 29]. Older People in Acute Care Survey (OPACS) is a tool that measures the attitude of hospital nurses regarding the care of the older people; it was developed in Australia and validated in the United States among 130 participants [25]. The tool was developed in the English language yet not translated into the local language because nurses are trained with English as a medium of instruction and in their working area they write and document patient records in English language. Then, the reliability of knowledge and attitude questions was checked by using Cronbach’s alpha 0.71 for knowledge and 0.89for attitude.

The data collection tool contains five sections. Section one includes Socio-demographics characteristics such as age, gender, marital status, religion, ethnicity, level of education, experience, and monthly income. Section two includes personal related factors such as lived with older people and caring for older people in clinical practice. Section three includes institutional related factors such as type of hospital, training regarding older people care, presence of guideline, working unit, and working hours. Section four includes a set of knowledge questions containing 30 true/false items. The overall nurses’ knowledge score towards the care of older people was obtained by recoding and converting every correct answer into 1 and incorrect answer into 0. Section five includes the attitude questions which consist of 34 items and are meant to measure the attitude of nurses towards the care of the older people. Items were answered with a five-point Likert scale (1. Strongly disagree, 2. Disagree, 3. Unsure,4. Agree, 5. Strongly agree).

### Data collection procedures

Data were collected using a pre-tested and structured self-administered questionnaire. Data were collected by eight BSc nurses and four MSc *nurses were hired as* supervisors. The principal investigator provided a two days training to data collectors and supervisors about the purpose of the study and how to fill the questionnaire. After briefly presenting the purpose of the study and the individual nurses in the study area, data collectors were responsible for the distribution and collection of the questionnaire.

### Data quality control and management

The questionnaire was pre-tested before the actual data collection on 22 (5%) nurses in *the* Holota primary hospital. Cronbach’s alpha was 0.87 and 0.89 for knowledge and attitude respectively, indicating adequate internal consistency. A two days training was given for data collectors and supervisors regarding the study, questionnaire, and data collection procedure. All data were checked for completeness and consistency on the data collection day, before and during analysis.

### Data processing and analysis

The data were coded and entered into epi-data version 4.6 and exported to SPSS version 25 for analysis. Descriptive statistics were carried out and summarized with texts, tables, and figures. Model fitness was checked by Hosmer and Lemeshow test of 0.48 for knowledge and 0.46 for attitude and it was fitted. Both bivariable and multivariable binary logistic regressions were used to assess the association between the outcome variables and the explanatory variables. Variables with a *p*-value less than 0.25 in bivariable logistic regression were fitted into the multivariable logistic regression model. Adjusted odds ratios were calculated and variables with a *p*-value less than or equal to 0.05 at 95% confidence interval were declared as significant to the outcome variables.

## Results

### Socio-demographic characteristics of the study participants

Out of the total sample size (423), 411 nurses participated in the study with a 97.16% response rate. The mean age of the participants was 29.11 (SD ± 3.84) years. More than half (55.2%) of the nurses were male. Most of the participants (88.1%) were BSc degree holders and above. Nearly half of the participants (44.8%) had 3–5 years of work experience. More than half of the participants (53.8%) were married and nearly half (47.7%) of the respondents had an income between 6200 and 8017 Ethiopian Birr. (Table 1).

**Table 1:** Socio-demographic characteristics of nurses working at public hospitals in West Shoa Zone, Ethiopia, 2021(*n* = 411)

Variables	Category	Frequency	Percentage
Age	20–25 years	84	20.4
	26–30 years	131	31.9
	> 30 years	196	47.7
Gender	Male	227	55.2
	female	184	44.8
Marital status	Single	190	46.2
	Married	221	53.8
Religion	Orthodox	117	28.5
	Muslim	80	19.5
	Protestant	184	44.8
	Wakefata	30	7.3
Ethnicity	Oromo	390	94.9
	*Others	21	5.1
Level of education	Diploma	49	11.9
	BSc and above	362	88.1
Year of experience	<=2 years	126	30.7
	3–5 years	184	44.8
	> 5 years	101	24.6
Monthly income (ETB)	< 6200	166	40.4
	6200–8017	196	47.7
	8018–9056	23	5.6
	> 9056	26	6.3

*Others = (Amhara, Tigre and Gurage)

### Personal and institutional related factors of study participants

More than half i.e.,51.8 and 54.6% of the respondents have lived with older people and ever cared for older people respectively. Similarly, 22.1% have been working in the outpatient department (OPD) and 36% of the participants were working in primary hospitals. (Table 2).

**Table 2:** Personal and institutional related factors of nurses working at public hospitals in West Shoa Zone, Ethiopia, 2021(*n* = 411)

Variables	Frequency	Percentage
Lived with older people		
Yes	213	51.8
No	198	48.2
Caring for older people in clinical practice		
Yes	224	54.6
No	187	45.4
Type of hospital working in		
Primary	148	36
General	136	33.1
Referral	127	30.9
Working unit/ward		
Medical ward	73	17.8
Surgical ward	66	16.1
Operation room	54	13.1
Emergency	52	12.7
Outpatients’ department	91	22.1
Adult intensive care unit	28	6.8
Gynecology ward	47	11.4
Working hours per week		
39–60 h.	338	82.2
> 60 h.	73	17.8

### Nurses’ knowledge toward the care of the older people

Overall, 153(37.2%) (95% CI: 33, 42%) of the respondents had good knowledge, whereas, 258 (62.8%) had poor knowledge toward the care of the older people. (Table 3).

**Table 3:** Nurses response on KOP-Q scale at public hospitals in West Shoa Zone, Ethiopia, 2021(*n* = 411)

Items	Correct answer	Incorrect answer
Forgetfulness, attention problems, and indecisiveness are all symptoms of aging, rather than indicators of depression	164(39.9%)	247(60.1%)
Urinary incontinence in an older person may indicate that the person is suffering from a urinary tract infection	333(81%)	78(19%)
Patients with a cognitive disorder, such as dementia, are at increased risk for delirium	349(84.9%)	62(15.1%)
Malnutrition may have a negative impact on a person’s ability to think	344(83.7%)	67(16.3%)
In general, older people are more sensitive to medication because their kidney and liver functions are declining	333(81%)	78(19%)
Meeting with families during patient assessment is only required for persons suffering from dementia	308(74.9%)	103(25.1%)
For older people, bed rest is important to enhance recovery	256(62.3%)	155(37.7%)
Patients rarely remember that they were restless during delirium	265(64.5%)	146(35.5%)
The elderly do not participate in physical activity, because they need less fluid	319(77.6%)	92(22.4%)
Asking patients whether they have fallen in the past six months is a good way of assessing increased risk of falling	315(76.6%)	96(23.4%)
Pressure ulcers can develop when the blood supply to tissue is stop for two hours	137(77.1%)	94(22.9%)
Depression is diagnosed more commonly in younger people than in older	322(78.3%)	89(21.7%)
Lowering the frequency of a medication is an effective intervention to achieve adherence by patients	305(74.2%)	106(25.8%)
Patients who are incontinent must get their soiled clothes changed, but they do not need to go to the toilet afterwards	290(70.6%)	121(29.4%)
It is good to have older people drink more often, because they have a reduced thirst sensation	275(66.9%)	136(33.1%)
In the case of delirium, bright lighting should be used to illuminate all of the corners of the room	241(58.6%)	170(41.4%)
Medication may cause geriatric problems such as memory deficits, incontinence, falling and depression	331(80.5%)	80(19.5%)
Overburdening of family caregivers may lead to abuse of the person for whom they are providing care	335(81.5%)	76(18.5%)
It is good to provide extensive instruction about how to complete tasks to patients suffering from apraxia	224(54.5%)	187(45.5%)
It is best to talk at a normal volume when listening to hearing-impaired older patients	284(69.1%)	127(30.9%)
An older person with a BMI of > 25 cannot be undernourished	280(68.1%)	131(31.9%)
In the case of difficulty swallowing, all medicines must be ground to ensure that patients ingest them	256(62.3%)	155(37.7%)
In the case of depression, memory problems may occur	315(76.6%)	96(23.4%)
Most family caregivers do not need additional support from home care services	259(63%)	152(37%)
As a nurse, you have to speak clearly into the ear of the hearing-impaired older patient	213(51.8%)	198(48.2%)
Pain medication should be administered to older people as little as possible, due to the possibility of addiction	210(51.1%)	201(48.9%)
We identify pressure ulcers only if blister formation have occurred	238(57.9%)	173(42.1%)
In the case of delirium, activities should be spread out evenly over the day	324(78.8%)	87(21.2%)
The risk of falling is higher for people in the hospital setting compared with those who are living at home	305(74.2%)	106(25.8%)
Stress incontinence may occur in patients who are not capable of opening their own trousers	270(65.7%)	141(34.3%)

### Nurses’ attitude towards care of older people

The result of this study revealed that 188(45.7%), (95% CI: 40.9, 50.6%) of the respondents had a favorable attitude whereas, 223(54.3%) had an unfavorable attitude toward the care of the older people. (Table 4).

**Table 4:** Nurses response on OPACS scale of attitude questions at public hospitals in West Shoa Zone, Ethiopia, 2021(*n* = 411)

Items	SD %	D %	U %	A %	SA %
Do you like to care for older patients	12.9%	6.8%	4.9%	30.7	44.8%
*Do you agree older patients are confused	39.4%	31.6%	7.8%	9%	12.2%
*Do you agree older patients pretend not to hear you	13.6%	11.9%	9.2%	21.7%	43.6%
*Older patients are a nuisance to care	12.2%	18%	10.7%	19.7%	39.4%
*Older patients are more likely to be depressed than younger patients	14.8%	12.7%	17.5%	31.6%	23.4%
*Older patients have to follow special diets	14.8%	10.5%	15.1%	30.4%	29.2%
*Older patients do not know the actions and interactions of their medications	25.3%	24.6%	16.3%	12.4%	21.4%
*Older patients require less pain-relieving mediation than younger patients	20.7%	12.2%	16.5%	27%	23.6%
*Older patients become addicted to sleeping medications easily	31.4%	35.3%	12.2%	5.6%	15.6%
*Incontinent patients are bothersome	17%	8%	15.3%	36.3%	23.4%
*Do you agree urinary incontinence is part of the aging	13.6%	5.6%	10.9%	40.1%	29.7%
Patients who are older are more worried with their bowel habits than those who are younger	13.9%	6.6%)	7.8%	38.2%	33.6%
Elder patients are uncomfortable when their bodies are exposed	16.3%	5.8%	8.5%	34.2%	34.8%
*Too many older patients receive life-sustaining care	38%	28.2%	10.5%	6.3%	17%
Older patients have more discharge problems than do younger patients	23.6%	5.4%	9%	30.2%	31.9%
At the time of discharge older patients are likely to be more dependent than younger patients	30.4%	6.3%	6.6%	32.4%	24.3%
Older patients require placement in long term care following a hospital admission	37.5%	5.6%	6.1%	29.7%	21.2%
*Older patients have extensive lengths of stay and take up beds that could be used for sicker patients	17.3%	25.1%	4.4%	9.5%	43.8%
*There are too many older patients in acute care hospitals	18.7%	23.6%	7.5%	8%	42.1%
It would be a good idea for all hospitals to have an acute geriatric unit	40.4%	4.9%	7.1%	22.9%	24.8%
Older patients are likely to be on more medication when admitted to the hospital than younger patients	40.1%	6.6%	8.3%	22.4%	22.6%
Older patients become confused in a new setting	38.2%	6.3%	10%	21.7%	24.8%
Older patients feel isolated in the acute care setting	37.5%	7.8%	10.7%	22.9%	21.2%
*In the hospital, eating and drinking are the most common activities performed by older patients	16.3%	27.7%	10.7%	6.8%	38.4%
Older patients have more skin problems than younger patients	35.8%	8%	8%	28.2%	20%
Older patients are more likely to require assistance with mobility than younger patients	36.5%	5.1%	7.8%	30.7%	20%
A lot of older patients have stiff joints	31.4%	7.8%	6.8%	30.2%	23.8%
Older patients tend not to tell health professional if they are incontinent	35.5%	10.7%	6.1%	27.3%	20.2%
Older patients experience changes in bowel elimination patterns in the acute care setting	34.5%	9.5%	6.1%	29.2%	20.7%
Older patients are more likely to have open surgical procedures than laparoscopic surgery	35.5%	9%	8%	24.6%	22.9%
Older patients become confused after operations/procedures	32.6%	5.4%	8.8%	25.1%	28.2%
Older patients are more likely to develop post-operative complications	33.8%	6.6%	5.8%	27.7%	26%
Older patients are particularly prone to nosocomial infections	30.9%	4.6%	8.5%	25.1%	30.9%
For older patients, early discharge is difficult to achieve	24.8%	7.1%	7.1%	26.5%	34.5%

*SD* Strongly Disagree, *D* Disagree, *U* Unsure, *A* gree and *SA* Strongly Agree

### Factors associated with nurses’ knowledge toward the care of older people

All independent variables were entered into the bivariable logistic regression model and variables with a *p*-value of < 0.25 were fitted into the multivariable logistic regression analysis. In multivariable logistic regression analysis age greater than 25 years and above, being BSc degree holder and above, work experience greater than two years and above, ever lived with older people, and nurses who were working in adult intensive care unit were significantly associated with knowledge towards the care of the elderly patients at a *p*-value of less than or equal to 0.05; 95% CI.

Nurses with the age of 26–30 years were 2.12 times more likely to have knowledge toward the care of the older people than those who are between ages of 20–25 years [AOR: 2.12; 95% CI;(1.03, 4.34)], and those participants whose age was > 30 years were 2.37 times more likely knowledgeable than those who are between ages of 20–25 years [AOR: 2.37; 95% CI: (1.18, 4.75)].

Those nurses who had 3–5 years of work experience were 2.22 times more likely knowledgeable than those who had less than or equal to 2 years of work experience [AOR: 2.22; 95% CI; (1.08, 4.56)], and participants who had > 5 years of work experience were 3.00 times more likely knowledgeable than those who had less than or equal to 2 years of experience [AOR: 3.00; 95% CI; (1.21, 7.41)].

In addition, nurses who had BSc degree and above were 3.57 times more likely knowledgeable than those nurses who had diploma [AOR: 3.57; 95 CI; (1.40, 9.09). (Table 5).

**Table 5:** Factors associated with nurse’s knowledge towards the care of the older people in bivariable and multivariable logistic regression at public hospitals in West Shoa Zone, Ethiopia, 2021 (*n* = 411)

Variables	Knowledge		COR (95% CI)	AOR (95% CI)	*P*-value
	Good	Poor			
Age					
20–25 years	15	69	1.0	1.0	
26–30 years	48	83	2.66(1.37, 5.15)	2.12(1.03, 4.34) *	0.040
> 30 years	90	106	3.90(2.09, 7.29)	2.37(1.18, 4.75) *	0.015
Work experience					
< =2 years	23	103	1.0	1.0	
3–5 years	71	113	2.81(1.63, 4.83)	2.22(1.08, 4.56) *	0.029
> 5 years	59	42	6.29(3.45, 11.47)	3.00(1.21, 7.41) *	0.017
Level of education					
Diploma	6	43	1.0	1.0	
BSc and above	147	215	4.90(2.03, 11.80)	3.57(1.40, 9.09) *	0.007
Ever lived with the older people					
Yes	101	112	2.53(1.67, 3.83)	2.14(1.34, 3.42) *	0.001
No	52	146	1.0	1.0	
Care of older people in clinical practice					
Yes	98	126	1.86(1.23, 2.81)	1.53(0.95, 2.46)	0.075
No	55	132	1.0	1.0	
Monthly income ETB					
< 6200	36	130	1.0	1.0	
6200–8017	87	109	2.88(1.81, 4.58)	1.10(0.57, 2.13)	0.756
8018–9056	14	9	5.61(2.25, 14.02)	1.65(0.49, 5.60)	0.415
> 9056	16	10	5.77(2.41, 13.82)	2.03(0.65, 6.28)	0.217
Working unit/ward					
MW	29	44	1.72(0.78, 3.80)	1.47(0.61, 3.51)	0.386
AICU	15	13	3.01(1.13, 8.03)	3.03(1.03, 8.91) *	0.043
OPD	40	51	2.05(0.95, 4.39)	1.56(0.67, 3.65)	0.299
EM	18	34	1.38(0.58, 3.26)	1.28(0.49, 3.31)	0.605
OR	17	37	1.23(0.50, 2.83)	1.18(0.45, 3.04)	0.731
SW	21	45	1.22(0.53, 2.77)	1.02(0.41, 2.54)	0.952
GynW	13	34	1.0	1.0	

*COR* Crude Odds Ratio, *AOR* Adjusted Odds Ratio, *1* Reference, *significant at *p*–value <=0.05, *CI* confidence interval

### Factors associated with nurses’ attitude toward the care of the older people

All independent variables were entered into the bivariable logistic regression model and variables that were < 0.25 were fitted into the multivariable analysis. In multivariable logistic regression analysis; being female, having BSc degree and above, ever lived with older people, and care of the older people in clinical practice were significantly associated with nurses’ attitude toward the care of the older people at *p*-value less than or equal to 0.05; 95% confidence interval.

Female nurses were 2.04 times more likely to have a favorable attitude when compared to their counterparts [AOR: 2.04, CI: (1.35, 3.12)]. Additionally, nurses who had a BSc degree and above were 2.77 times more likely to have a favorable attitude when compared to those who had a diploma [AOR: 2.77, CI; (1.35, 5.65)]. (Table 6).

**Table 6:** Factors associated with nurse’s attitude towards the care of the older people in bivariable and multivariable logistic regression at public hospitals in West Shoa Zone, Ethiopia, 2021 (*n* = 411)

Variables	Attitude		COR (95% CI)	AOR (95% CI)	*P*-value
	Favorable	Unfavorable			
Gender					
Female	103	81	2.12(1.42, 3.15)	2.04(1.35, 3.12) *	0.001
Male	85	142	1.0	1.0	
Marital status					
Married	112	109	1.54(1.04, 2.28)	1.37(0.90, 2.10)	0.136
Single	76	114	1.0	1.0	
Level of education					
Diploma	13	36	1.0	1.0	
BSc & above	175	187	2.59(1.33, 5.04)	2.77(1.35, 5.65) *	0.005
Lived with older people					
Yes	116	97	2.09(1.40, 3.10)	1.59(1.03, 2.44) *	0.035
No	72	126	1.0	1.0	
Care of the older people in clinical practice					
Yes	116	108	1.71(1.15, 2.54)	1.63(1.06, 2.53) *	0.026
No	72	115	1.0	1.0	
Knowledge towards the care of older people					
Good	79	74	1.45(0.97,2.18)	1.13(0.72,1.78)	0.569
Poor	109	149	1.0	1.0	
Working unit/ward					
MW	29	44	1.27(0.59, 2.74)	1.19(0.53, 2.67)	0.671
AICU	11	17	1.25(0.47, 3.30)	1.15(0.41, 3.25)	0.779
OPD	48	43	2.16(1.04, 4.48)	1.90(0.87, 4.12)	0.105
EM	20	32	1.21(0.53, 2.75)	1.30(0.54, 3.10)	0.547
OR	30	24	2.42(1.08, 5.43)	2.20(0.93, 5.22)	0.072
SW	34	32	2.05(0.95, 4.45)	2.06(0.90, 4.69)	0.084
GynW	16	31	1.0	1.0	

*COR* Crude Odds Ratio, *AOR* Adjusted Odds Ratio, *1* Reference, *Significant at *p*-value<=0.05, *CI* confidence interval

## Discussion

In this study, the overall good knowledge of nurses towards the care of older people was found to be 37.2% (95% CI: 33, 42%). The finding of this study was higher than the study conducted in *Bangladesh, 32.8%* [13]*.* This variation could be because of the difference in the study setting; more than half of the respondents in Bangladesh had a diploma level of education. *On the contrary,* the finding was lower than a study conducted *in* Nigeria, 96% *[*30*]*. The possible reason might be the difference in study participants. The implication of the finding revealed the variability of nurses’ knowledge about the older people care in different settings. As studies demonstrated, care for older people is not considered a very attractive area of nursing practice [31, 32]; as a result, there may be professional disrespect for choosing to work with older people [8]. Thus, having good knowledge might attract and motivate nurses to work with the older people.

In this study, age was significantly associated with good knowledge towards the care of older people. Nurses whose age group is 26–30 years were 2.12 times more likely to have good knowledge towards the care of older people compared to those nurses aged between 20 and 25 years. Furthermore, those participants whose age was > 30 years were 2.37 times more likely to have good knowledge towards the care of older people compared to those whose age group is 20–25 years. The finding was supported by a study conducted in Korea [22]. This could be because nurses with higher age have more experience caring for older people, which enhances their knowledge regarding the older people. Older nurses increased their clinical thinking ability because of their experiences in caring for the older people as well as exposure to more complicated cases [33]. Thus, to enhance the quality of care for the older people, junior nurses better work with the senior staffs and should share their experience.

The work experience was found to be the determinant factor of the knowledge of nurses towards the care of older people. Nurses who had 3–5 years of work experience were 2.22 times more likely to have good knowledge towards the care of older people compared to those who had less than or equal to 2 years of working experience. Similarly, nurses who had more than 5 years of experience were 3 times more likely to have good knowledge towards the care of older people compared to those nurses with less than or equal to 2 years of working experience. The result of this study is complemented by a study conducted in Korea [34]. Nurses with more experience might have a better chance of eventually gaining access to up-to-date knowledge about the care of older people through their daily observations and practices. This implies the great importance of work experience to have a good knowledge and would possibly help for a better evidence-based practice.

The findings of this study revealed that the level of education is significantly associated with the knowledge of nurses toward the care of the older people. Nurses who had a baccalaureate degree and above were 3.57 times more likely to have good knowledge towards the care of older people compared to those having a diploma. The result of this study is supported by a study conducted in the Netherlands [23]. This is because education improves the knowledge of nurses towards the care of patients. Higher education curriculum helps nurses to get the chance of participating in different seminars, workshops, reviewing different kinds of literature and updating themselves. Nurses who have a higher educational status are more likely to protect their patients’ health and cope with changes in their mental and physical abilities, so older people can stay independent and active as long as possible https://explorehealthcareers.org/career/geriatrics/geriatric-staff-nurse/. Thus, nurses should be motivated to engage in their professional development and the educational carrier. This might help for a better older people care.

The findings of this study revealed that nurses who were working in the intensive care unit were significantly associated with knowledge toward the care of older people. Nurses who were working in the adult intensive care unit were 3.03 times more likely to have good knowledge of the care of older people compared to those working in the gynecology ward. The result of this study contradicts the study done in Portugal [17]. This could be due to the fact that as the nurses frequently contacted with the patients, the nurses have developed a good knowledge towards the care of elderly patients. The finding implies that nurses working in different working units would have a better knowledge and this might be important for a better evidence-based practice on the older people care.

In this *study, 45.7%* (95% CI: 40.9, 50.6%) of the respondents had a favorable attitude concerning the care of older people. This finding was higher than a study done in Iran *9.8%* *[*11*]*. The possible justification could be the type of tool used, the study period, and the difference in working experience. On the other hand, the finding was lower than the study done in Bangladesh, 63.8% [13]. The variation could be because of the difference in the socio-demographic characteristics, socioeconomic status, and taking of geriatric care training. Positive attitudes towards the older people care are highly needed and critically important for better healthcare and wellbeing of the older people [16].

The sex of the study participants was significantly associated with nurse’s attitudes towards the care of older people. Female nurses were 2.04 times more likely to have a favorable attitude towards the care of older people compared to those male counterparts. This finding is supported by a study done in Iran [11]. This could be for the reason that females have better concentration on their work and they are naturally gifted in caring behaviors. For good quality care of elderly patients, both male and female nurses should work strongly to improve care and special attention should be given for male nurses.

This study revealed a significant association between the levels of education and attitude of nurses towards the care of the older people. Nurses who had a BSc degree and above were nearly three times more likely to have a favorable attitude towards the care of older people compared to those who had a diploma. The result of this study is complemented by a study conducted in Nepal [35]. This could be because an increased level of education helps to read different kinds of literature regarding the care of the older people, which will bring a favorable attitude. To have a favorable attitude towards a better quality of older people care, nurses with a lower educational status should get short- and long-term training.

Nurses who had lived with the older people were significantly associated with nurses’ attitude towards the care of the older people. Nurses who had lived with the older peoplewere 1.6 times more likely to have a favorable attitude compared to those who didn’t live with the older people. The result of this study is complemented by a study conducted in Korea [34]. This could be because nurses who have lived with the older people might help the older people and appreciate their problems. This might result in a positive attitude towards care for elderly people.

This study also investigated nurses who had an experience of the care of older people in clinical practice and found out that it significantly associated with nurses’ knowledge regarding the care of the older people. Nurses who had experienced the care of the older people in clinical practice were 1.63 times more likely to have a favorable attitude towards the care of older people compared to those who did not have the experience of caring for the older people in clinical practice. The result of this study is supported by a study conducted in Turkey [20]. The possible justification might be that engaging with older people helped the nurses understand problems of the elderly which motivated them to care for the older people.

### Strength and limitations

This study focused on one of the neglected special population groups and possibly it could be the first study in the study area and could have added a valuable contribution to the medical field. However, it has some limitations. Firstly, since self-administered questionnaire for knowledge and attitude regarding care of the older people was used, there may be recall bias. Secondly, lack of comparable studies in Ethiopia made the local comparison and discussion difficult. Thirdly, nurses working in the pediatrics units were not included in the study.

## Conclusion

According to this study, less than half of the study participants had good knowledge and a favorable attitude toward the care of older people. The study also showed that age greater than 25 years and above, serving for greater than two years and above, being BSc degree holder and above, ever lived with the older people, and nurses who were working in adult intensive care unit were significantly associated with the knowledge of nurses. Moreover, being female, being BSc degree holder and above, ever lived with the older people, and care of older people in clinical practice were significantly associated with nurses’ attitude towards care of the older people. Likewise, continuous professional development regarding the care of the older people is important to enhance nurses’ knowledge and attitude.

## Data Availability

The summary data are available in the main document**.** The datasets used and/or analyzed during the current study are available from the corresponding author on reasonable request.

## Abbreviations

AOR: Adjusted odds ratio
CI: Confidence interval
COR: crude odds ratio
ICU: Intensive care unit
KOP-Q: Knowledge of older patient’s quiz
OPACS: Older patients in acute care survey
OPD: Outpatient department
SPSS: statistical package for social sciences
